# A dual-functional PEG-tyrosine hydrogel with photothermal effect and antioxidant capacity for cancer therapy and tissue regeneration

**DOI:** 10.1093/rb/rbag034

**Published:** 2026-03-04

**Authors:** Haitao Zhu, Yunfan Shen, Zhebin Wu, Yide Yi, Yuanfei Chen, Wenjin Xu, Xin Wang, Hsiang-I Tsai, Dongqing Wang, Xiang Liao, Yanfang Liu, Qinghua Li

**Affiliations:** Institute of Medical Imaging and Artificial Intelligence, Jiangsu University, Zhenjiang 212001, China; Department of Medical Imaging, The Affiliated Hospital of Jiangsu University, Zhenjiang 212001, China; Institute of Medical Imaging and Artificial Intelligence, Jiangsu University, Zhenjiang 212001, China; Institute of Medical Imaging and Artificial Intelligence, Jiangsu University, Zhenjiang 212001, China; Institute of Medical Imaging and Artificial Intelligence, Jiangsu University, Zhenjiang 212001, China; Institute of Medical Imaging and Artificial Intelligence, Jiangsu University, Zhenjiang 212001, China; Institute of Medical Imaging and Artificial Intelligence, Jiangsu University, Zhenjiang 212001, China; Institute of Medical Imaging and Artificial Intelligence, Jiangsu University, Zhenjiang 212001, China; Institute of Medical Imaging and Artificial Intelligence, Jiangsu University, Zhenjiang 212001, China; Department of Medical Imaging, The Affiliated Hospital of Jiangsu University, Zhenjiang 212001, China; Institute of Medical Imaging and Artificial Intelligence, Jiangsu University, Zhenjiang 212001, China; Department of Medical Imaging, The Affiliated Hospital of Jiangsu University, Zhenjiang 212001, China; Institute of Medical Imaging and Artificial Intelligence, Jiangsu University, Zhenjiang 212001, China; Department of Central Laboratory, Affiliated People’s Hospital of Jiangsu University, Zhenjiang 212001, China; Institute of Medical Imaging and Artificial Intelligence, Jiangsu University, Zhenjiang 212001, China; Department of Medical Imaging, The Affiliated Hospital of Jiangsu University, Zhenjiang 212001, China

**Keywords:** polypeptide hydrogel, photothermal therapy, ROS scavenger, wound healing

## Abstract

Achieving optimal tumor eradication while minimizing off-target toxicity and enhancing patient recovery remains a central challenge in translational oncology. Traditional therapies such as radiotherapy and chemotherapy are associated with systemic toxicities and resistance, compromising outcomes. Photothermal therapy provides spatial targeting and a minimally invasive approach but risks collateral tissue damage, particularly to skin. We report an injectable, biodegradable PEG-tyrosine hydrogel with oxidative modification (PETyrO) designed for dual-function tumor photothermal therapy and post-treatment wound regeneration. PETyrO is prepared in two steps: (i) ring-opening polymerization of L-tyrosine N-carboxyanhydride (Tyr-NCA) monomers, followed by (ii) enzymatic oxidation using tyrosinase. The hydrogel forms at a final concentration of 100 mg/mL before injection. Owing to its phenolic groups, PETyrO exhibits pronounced reactive oxygen species (ROS) scavenging capacity, promoting epithelial regeneration at treatment sites. In summary, the melanin-like, biocompatible system shows minimal cytotoxicity, achieves a photothermal conversion efficiency of 36% at 808 nm and integrates ROS scavenging with wound-healing properties. More importantly, unlike traditional dopamine-based hydrogels, this peptide hydrogel features a well-defined structural framework and can degrade gradually *in vivo*. Through sequential control, it achieves tunable and repeatable structures and mechanical properties, increasing its potential for clinical translation. This multifunctional biomaterial offers a new paradigm for dual therapeutic capabilities.

## Graphical abstract

**Graphical Abstract:**
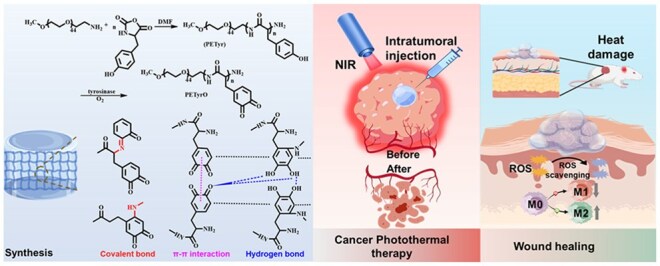

## Introduction

Cancer remains a leading global public health burden. The World Cancer Report estimates 20.0 million incident cancer cases worldwide in 2022, corresponding to an estimated case fatality rate of 48.5% [1]. Conventional therapies, including surgical resection, radiotherapy and chemotherapy, can adversely affect healthy tissues and vital organs. In particular, high-dose chemotherapeutic regimens exacerbate systemic toxicity [2–5]. An urgent need exists to develop innovative therapeutic strategies for malignant tumors. Photothermal therapy (PTT), a spatially targeted and minimally invasive modality employing photothermal agents (PTAs), induces localized hyperthermia through near-infrared irradiation (NIR) to selectively eradicate tumor tissue and has demonstrated promising efficacy in preclinical and clinical studies [6–8]. PTAs developed to date comprise both inorganic and organic nanomaterials. Inorganic PTAs, such as Au- [9, 10], Ag- [11], Pd-based nanoparticles [12] and carbon-based nanomaterials [13], demonstrate high photothermal stability under sustained light exposure, but their slow degradation can raise bioaccumulation risks and long-term biosafety concerns. Indocyanine green (ICG), an organic NIR fluorescent dye approved by the U.S. Food and Drug Administration (FDA), demonstrates superior photothermal conversion efficiency and is considered a high-performance agent for photothermal therapy in clinical applications [14, 15]. However, photobleaching and rapid degradation undermine photothermal stability, a critical factor in achieving optimal therapeutic efficacy [16, 17]. Furthermore, intravenous administration of these PTAs may result in off-target accumulation, potentially causing adverse effects in healthy organs [18, 19]. Consequently, novel biomedical PTAs exhibiting high photothermal conversion efficiency, enhanced thermal stability and biodegradability are urgently needed. Moreover, local thermotherapy may induce excessive production of reactive oxygen species (ROS), potentially impairing repair of thermal damage *via* ROS-mediated mechanisms under elevated local temperatures [20–22]. Therefore, strategies that simultaneously eradicate tumor cells and mitigate excess ROS are essential.

Melanin, a biopolymer with absorption extending into the near-infrared region, is ubiquitously distributed across almost all living organisms [23, 24]. Accordingly, biomaterials that integrate melanin-like architectures with phenolic components may enable photothermal therapy as well as post-treatment management of thermal injury. Polydopamine (PDA), an artificial melanin, has demonstrated substantial PTT capabilities and ROS-scavenging. Nevertheless, PDA hydrogels generally suffer from limited structural definition, uncertain degradability and batch-to-batch variability due to their stochastic polymerization chemistry. Tyrosine serves as an essential precursor in melanin biosynthesis and is oxidized *in vivo* by tyrosinase. This pigment is synthesized through enzymatic reactions catalyzed by tyrosinase, which oxidizes tyrosine *in vivo* [25, 26]. Meanwhile, as an aromatic polar α-amino acid bearing a phenolic hydroxyl moiety, tyrosine can effectively neutralize excess ROS generated by hyperthermia [27–29]. Therefore, tyrosine-based polymers are oxidized by tyrosinase to generate a melanin-like pigment that exhibits photothermal conversion efficiency comparable to natural melanin and shows promise for *in vivo* tumor therapy. Additionally, by reducing ROS, it facilitates wound healing in heat-damaged tissue, highlighting a protective function under thermal stress.

**Figure 1:**
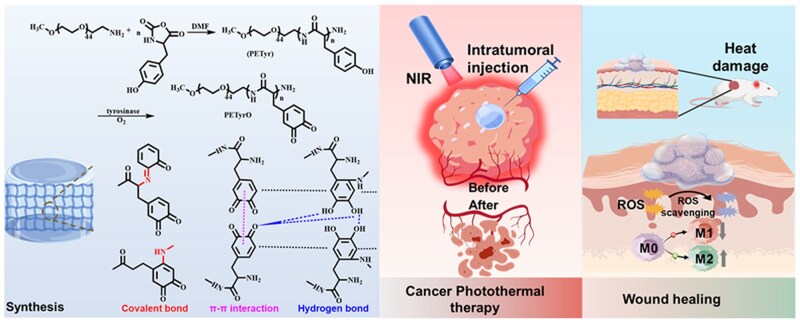
A schematic illustration of an injectable polypeptide hydrogel with dual photothermal therapy applications for cancer treatment and wound repair, serving as a versatile platform.

Building on this concept, a dual-functional hydrogel derived from tyrosine, possessing photothermal and ROS-scavenging properties, has been developed. The biomaterial undergoes oxidation to mimic melanin’s structure, enabling effective *in vivo* cancer treatment. The polyethylene glycol-tyrosine polymer (PETyr) was synthesized by ring-opening polymerization (ROP) using PEG-NH_2_ as the macromolecular initiator [30, 31], and is oxidized to form a melanin-like photothermal material (PETyrO) for tumor eradication. Furthermore, the phenolic moieties in PETyrO provide ROS scavenging after treatment. Compared with standard PDA hydrogels, PETyrO provides (i) a sequence-defined backbone enabling enzymatic and predictable degradation into amino acids, (ii) tunable, reproducible structure and mechanics via sequence control without sacrificing photothermal and ROS-scavenging performance. Collectively, characteristics render this hydrogel superior to the standard PDA system in biocompatibility, biodegradability, reproducibility and translational potential. It also features dual functionality: strong photothermal performance and enhanced tissue regeneration capability. Collectively, this multifunctional system offers new strategies for designing biomaterials with innovative architectures and broad therapeutic potential (Figure 1).

## Materials and methods

### Materials

mPEG-NH_2_ (Mn = 2000 g/mol) was purchased from MeloPEG Technology Co., Ltd. L-tyrosine N-carboxy anhydrides (L-Tyr NCAs) were custom-made by Chengdu Enlai Biological Technology Co., Ltd. Tyrosinase and N, N-dimethylformamide (DMF) were purchased from Sigma-Aldrich.

### The synthesis and characterization of PETyrO hydrogel

Following previously reported protocol, PETyr copolymers were synthesized by ROP of L-Tyr NCAs using mPEG-NH_2_ as the macromolecular initiator, as illustrated in Figure 1 [32, 33]. The molar ratio of mPEG_2000_-NH_2_ initiator to L-Tyr NCAs was 1:8. Polymerization was conducted in anhydrous DMF at 40°C for 72 h, yielding PETyr block copolymers that were isolated as white powders after dialysis and lyophilization. PETyrO was subsequently synthesized by enzymatic oxidation of PETyr (stock: 1 mL of PETyr copolymer at 1 mM) using 0.1 mL of tyrosinase (200 U/mL) over 48 h, followed by incubation at 60°C for 1 h to inactivate the enzyme. The PETyrO product was recovered by dialysis and freeze-drying. Prior to use, the hydrogel was formed at a final concentration of 100 mg/mL. Secondary structures were analyzed by FTIR spectra recorded on an IR Prestige 21 spectrometer (Shimadzu) and CD spectra were acquired with a J-810 spectropolarimeter (JASCO). All measurements were conducted on freeze-dried copolymer samples with an AVATAR 360 spectrometer.

### Effect of oxidation on the gelation behavior

The sol-gel transition was assessed by the vial inversion method. Copolymer solutions (∼1 mL, 100 mg/mL) were prepared in 3 mL glass vials, and samples were deemed gels if no flow occurred within 1 min after inversion. Rheological analysis was performed using an AR 200ex rheometer (TA Instruments). Frequency sweeps from 0.1 to 100 rad/s were conducted at 25°C under 1.0% strain to determine the storage modulus G′ and loss modulus G″. Temperature ramp tests from 20 to 60°C at 0.5°C/min were carried out under 1.0% strain and 1.0 rad/s to monitor G′ and G″ during thermal variation. Secondary structural changes of the copolymers under thermal variation were analyzed by circular dichroism (CD) spectroscopy. Temperature-dependent CD spectra were recorded with a JASCO J-810 spectropolarimeter, using 0.1 mg/mL samples, 20–60°C, a heating rate of 1°C/min and 185–300 nm detection with 15 min of equilibration prior to measurement. The assembly morphology and internal microstructure of the hydrogel were characterized by scanning electron microscopy (SEM, Hitachi S-480).

The self-healing and injectability of the hydrogels were examined. Strain-amplitude sweep (γ = 10–90%) was performed to identify the critical strain of the PETyr and PETyrO hydrogels. The storage (G′) and loss (G″) moduli of both hydrogels were measured over four cycles at 10% and 90% oscillatory strain with a fixed frequency of 1 Hz at 37°C. Injectability was assessed by measuring the viscosity under a shear-rate sweep (37°C, 0.01–1000 1/s).

### Assessment of photothermal effect of PETyrO hydrogel *in vitro*

The photothermal effect was evaluated by dissolving copolymers in deionized water at 100 mg/mL and irradiating with an 808-nm NIR laser at power densities of 0.2, 0.6, 1.0, 1.5 and 2.0 W/cm^2^ for 5 min, respectively. To assess *in vitro* photothermal stability, samples underwent six consecutive irradiation cycles (808 nm, 2.0 W/cm^2^, 5 min per cycle) followed by natural cooling to ambient temperature.

### Cytotoxicity analysis

The cytotoxicity of PETyrO copolymer against NIH-3T3 and 4T1 cells was assessed *in vitro* using the Cell Counting Kit-8 (CCK-8) assay according to standard protocols. All experiments were performed in triplicate and results are reported as mean values ± standard deviation (SD). Fluorescence signals were detected at 494/517 nm (excitation/emission) for Calcein-AM and 528/617 nm (excitation/emission) for ethidium homodimer-1.

### Evaluation of photothermal therapy effect of PETyrO hydrogel materials *in vivo*

Prior to applying this hydrogel for cancer therapy, its blood compatibility and *in vivo* safety were evaluated. Blood compatibility was evaluated *via* a hemolysis assay. Fresh rabbit red blood cells (RBCs) were prepared into a suspension (4%) and exposed to varying concentrations of test polymers (PETyr, PETyrO), with negative controls (PBS) and positive controls (0.2% Triton X-100). After incubation at 37°C for 2 h, samples were centrifuged at 2000 rpm for 5 min, and the appearance of the red blood cells in the tubes was observed to assess hemolysis. *In vivo* safety was assessed *via* subcutaneous implantation (100 μL). All animal procedures were conducted in strict compliance with the Committee on the Use of Live Animals for Teaching and Research of the Jiangsu University. Experimental protocols were approved by Institutional Animal Care and Use Committee of Jiangsu University (Approval No. UJS-IACUC-AP-2024030135). Four weeks after implantation, tissue samples from major organs (heart, liver, spleen, lungs, kidneys) and from the skin were collected for histological staining to assess tissue changes.

The antitumor efficacy of PETyrO hydrogels was evaluated in female BALB/c mice bearing subcutaneous 4T1 tumors (syngeneic model, approximate volume ≈ 100 mm³). Mice were randomly assigned to six groups (*n* = 6): (i) PBS administered peritumorally, (ii) 808 nm NIR laser irradiation alone (1.5 W/cm^2^, 5 min), (iii) PETyr hydrogel administration, (iv) PETyrO hydrogel administration, (v) PETyr hydrogel + NIR irradiation and (vi) PETyrO hydrogel + NIR irradiation. The injection dose for each mouse was 100 μL around the tumor. Tumor dimensions and body weight were recorded every 48 h. Tumor volume was calculated as V = 1/2 × L × W^2^, where L is the longest diameter and W is the shortest perpendicular diameter. Following treatment, all animals were sacrificed for necropsy with collection of major organs (heart, liver, spleen, kidneys, lungs) and tumor tissues for histopathological evaluation. Collected tissues were formalin-fixed, paraffin-embedded and sectioned at 4–5 μm. Standard hematoxylin and eosin (H&E) and TUNEL staining protocols were implemented. Imaging analyses were conducted using digital microscopy (Axio Scan.Z1, Zeiss) and confocal laser-scanning microscopy (LSM 980, Zeiss) with ZEN imaging software. This comprehensive analysis assessed both PTT efficacy and potential systemic toxicity.

### Evaluation of ROS scavenging ability and antioxidation *in vitro*

The H_2_O_2_ scavenging capacity of PETyr and PETyrO hydrogels was quantified using the Amplite™ Hydrogen Peroxide Assay Kit (AAT Bioquest, USA) according to the manufacturer’s protocol by monitoring the decrease in H_2_O_2_ concentration. A standard calibration curve was generated for H_2_O_2_ concentrations in the range of 0–40 μM, and sample concentrations were determined from this curve.

The hydrogel’s free radical scavenging capacity was further analyzed using an established DPPH radical scavenging assay. PETyr and PETyrO solutions (1 mg/mL) were each added to 2 mL of DPPH ethanol solution (100 μg/mL). An ethanol solution without test samples served as the negative control. Following 30 min of incubation at room temperature, the absorbance of the reaction mixture was recorded at 515 nm using a Varioskan Flash microplate reader (Thermo Fisher Scientific).

To investigate the protective effects of PETyrO (1 mg/mL) on cellular oxidative stress, RAW 264.7 cells were treated with the compound. Cells were seeded in 96-well plates at a density of 1 × 10^4^ cells per well and exposed to 100 μM H_2_O_2_ to induce oxidative stress, resulting in elevated ROS production. Cellular viability was subsequently assessed using the CCK-8 assay. The concentrations of TNF-α and IL-1β in the culture medium were quantified using a commercial ELISA kit (Cusabio, Wuhan, China).

### *In vitro* migration assay

To examine the effects of PETyrO on HUVEC migration, a wound healing assay was conducted. HUVECs were plated in 24-well plates at a density of 5 × 10^4^ cells/well and incubated for 24 h to reach confluence. Following confluence attainment, the medium was aspirated and standardized wounds were generated using sterile 200-μL pipette tips. The cells were washed twice with PBS and treated with PETyrO-containing medium (1 mg/mL) for 6 h. Cellular migration was quantitatively analyzed after 6-h incubation using an inverted phase-contrast microscope (Leica DMI 300B, Germany).

### *In vitro* macrophage polarization

Bone marrow-derived macrophages (BMDMs) were isolated from C57BL/6 mice and cultured for 7 days according to established protocols [34]. Macrophages were primed with 100 ng/mL LPS for 24 h, followed by treatment with PETyrO. After 48 h, cells were harvested by centrifugation (1000 rpm, 5 min) and washed twice with ice-cold PBS. Cells were stained with FITC-anti-CD80, PE-F4/80 and APC-CD206 antibodies according to manufacturer’s protocols analyzed by flow cytometry using a BD Accuri C6 (BD Biosciences, USA). mRNA levels of IL-1β, inducible nitric oxide synthase (iNOS), vascular endothelial growth factor (VEGF) and CD206 were quantified using qRT-PCR. Protein expression of NF-κB p65 and HIF-1α was assessed by Western blotting. Total protein lysates were extracted using RIPA buffer (Beyotime, P0013B) supplemented with 1 mM PMSF protease inhibitor (Beyotime, ST506).

### *In vivo* wound healing performance of burnt model caused by photothermal therapy

The therapeutic efficacy of PETyrO hydrogel was evaluated in both an *in situ* model of photothermal injury and a burn injury model. The complex *in situ* photothermal wound model was conducted on the same mouse after photothermal treatment to ensure that the wounds observed post-PTT were those resulting from the tumor experiment. The mice in the PETyrO (+) group that underwent photothermal treatment were randomly assigned to two subgroups: PETyrO dressing treatment on the skin surface or no additional treatment (control). Wound progression was monitored through serial measurements with photographic documentation at Days 0, 3 and 8. Histological evaluation using H&E staining was performed to characterize tissue regeneration.

Since the injected hydrogel was located near the tumor tissue beneath the skin, while our wound dressing was used for the epidermal layer. As time goes on and with the application of NIR irradiation in tumor treatment, there may be differences in the retention amount of the hydrogel. In order to eliminate the influence of the previously injected hydrogel, standardized heat-induced burn models with 0.8 cm diameter circular wounds were established. The hydrogel was topically applied as a wound dressing to the affected areas, without the use of additional irradiation. Wound progression was monitored through serial measurements with photographic documentation at Days 0, 3, 7 and 14. Histological evaluation using H&E and Masson’s trichrome staining was conducted.

Immunofluorescence staining was performed on tissue sections to assess angiogenesis and inflammatory responses. After dewaxing and rehydration, antigen retrieval was conducted in citrate buffer using microwave heating. Autofluorescence quenching was then performed, and sections were mounted with a hydrophobic barrier. Following PBS washes, sections were blocked with 10% goat serum for 2 h at room temperature. They were incubated overnight at 4°C with primary antibodies: mouse anti-CD68 (1:200; Abcam) and rabbit anti-CD86 (1:200; Bioss). After overnight incubation, sections were washed three times (5 min each) with PBS (pH 7.4) under gentle agitation. After drying, sections were incubated for 1 h at room temperature with species-matched fluorescent secondary antibodies. Subsequent PBS washes (pH 7.4) were performed under agitation as described. Nuclei were counterstained with DAPI (0.1 μg/mL) for 10 min, followed by three PBS washes. Coverslips were mounted with anti-fade mounting medium, and images were acquired with a fluorescence microscope. Quantification was performed using ImageJ (v1.53).

### Statistical analysis

Statistical significance was assessed using *t*-test for comparisons between two groups and one-way ANOVA for comparisons among multiple groups. Data are presented as mean ± SD. Statistical significance thresholds are indicated as *P < 0.05, **P < 0.01 and ***P < 0.001.

## Results and discussion

### Synthesis and characterization of PETyrO hydrogel

**Figure 2:**
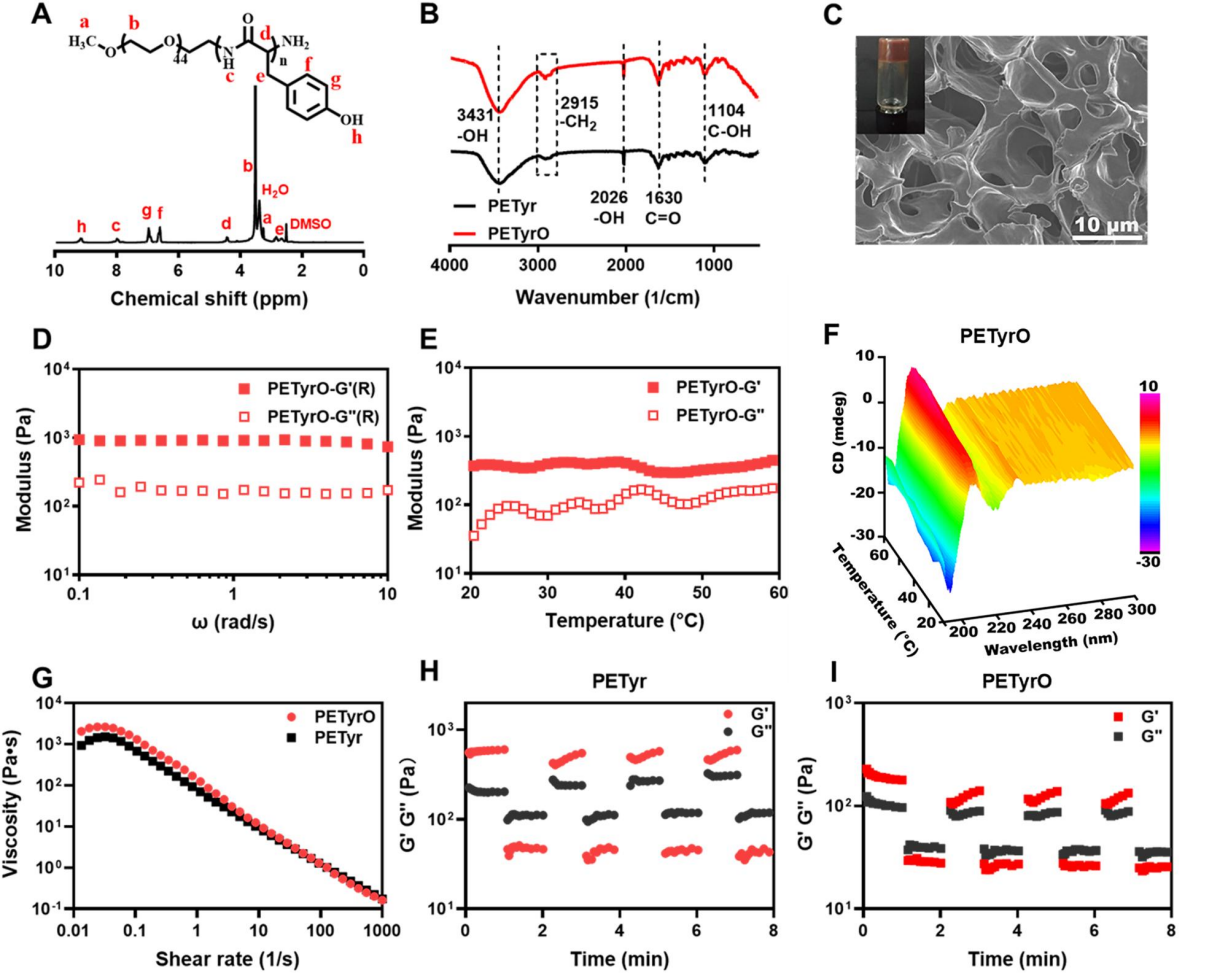
Synthesis and characterization of PETyrO polymer. (**A**) The ^1^H NMR, (**B**) FTIR spectral analysis. (**C**) SEM micrographs illustrating the interior microstructure of PETyrO hydrogel. (**D**) Viscoelastic properties of PETyrO hydrogel: Elasticity modulus (G′) and viscosity modulus (G″) of PETyrO hydrogel. (**E**) Temperature-dependent rheological behavior of PETyrO hydrogel. (**F**) The CD spectra of PETyrO hydrogel. (**G**) Viscosity vs shear rate profile of PETyr hydrogel and PETyrO hydrogel at 37°C. Strain-step test with strain altering between 10% and 90% for four cycles at 37°C of PETyr hydrogel (**H**) and PETyrO hydrogel (**I**).

Compared with conventional hydrogels, peptide hydrogels more closely mimic key properties of natural extracellular matrix components. These materials have been employed in diverse biomedical applications, including tissue-engineering scaffold fabrication and controlled drug delivery systems. The structural advantages of peptide hydrogels include biocompatibility, biodegradability and bioactivity, along with functional diversity and distinctive secondary structures such as α-helices and β-sheets [35, 36]. The PETyr polypeptide hydrogel was synthesized by ROP of L-Tyr NCA monomers, initiated by mPEG_2000_-NH_2_ as the macromolecular initiator. The block-length ratio between mPEG_2000_-NH_2_ macroinitiator and L-Tyr NCA monomers was systematically varied by adjusting the feed ratio. The lyophilized PETyr polymer was obtained as a free-flowing white powder. Experimental optimization demonstrated that a feed ratio of 1:8 (mPEG_2000_-NH_2_: L-Tyr NCA) achieved optimal hydrogel formation [31]. Figure 2A displays the ^1^H NMR spectra of PETyr. Characteristic resonances at 3.51 ppm (peak g) and 7.0–6.5 ppm (peaks b and c) correspond to the -O-CH_2_-CH_2_-O moiety in PEG and to aromatic protons in L-Tyr NCA, respectively. These diagnostic signals were identified in the ^1^H NMR spectrum of PETyr, confirming successful copolymer formation. PETyrO was subsequently obtained *via* enzyme-mediated oxidation of PETyr using tyrosinase. The FTIR spectrum (Figure 2B) revealed a prominent amide I band at 1630 cm^−1^, while the CD spectrum (Supplementary Figure S1A) exhibited a minimum at 220 nm. These spectral features collectively indicate that the tyrosine block adopts a conformation characterized by coexisting α-helix and β-sheet structures [37]. The results demonstrate that oxidation does not alter the secondary structure or self-assembly behavior in an aqueous solution.

Oxidative stability of hydrogels is crucial for their prospective applications. Therefore, we investigated the effect of oxidation on hydrogel formation. Hydrogel formation was first assessed by the vial-inversion method, and the resulting 3D porous structure was characterized by SEM imaging (Figure 2C and Supplementary Figure S1B). As shown in Supplementary Figures S1C and S2D, mechanical measurements revealed that the storage modulus (G′) of the hydrogels consistently exceeded the loss modulus (G″), particularly for samples subjected to six heating cycles, indicating preserved structural integrity after oxidative treatment. This study focuses on developing PETyrO hydrogel as a multifunctional platform for photothermal therapy, leveraging its intrinsic melanin-mimetic nanostructure [38, 39], with temperature-dependent effects on rheological properties and protein secondary structures examined. As shown in Figure 2E, G′ exceeded G″ between 20°C and 60°C, indicating a temperature-stable hydrogel network. Supplementary Figures S1D and S2F, show no significant change in secondary structure from 20°C to 60°C for either group, suggesting that temperature did not alter the secondary structure. Collectively, oxidation did not significantly alter the storage modulus (G′) or the loss modulus (G″) of the PETyrO hydrogel, demonstrating preserved rheological properties and structural integrity. Thermal analyses revealed stable rheological behavior and conserved secondary structure (α-helix and β-sheet) across the tested range (20–60°C), supporting the thermal stability of the oxidized PETyrO hydrogel and its suitability for photothermal applications.

Hydrogels with injectability and self-healing properties offer great convenience for *in vivo* biomedical applications. The injectability and self-healing ability of both hydrogels were assessed rheologically. First, as shown in Figure 2G, the viscosity of both hydrogels decreased with increasing shear rate, displaying typical shear-thinning behavior and hence good injectability. Subsequently, a continuous alternating strain sweep was conducted to evaluate the self-healing capacity (Figure 2H and I). The hydrogels underwent a transition from a gel network to a liquid state above a critical strain due to rupture of physical crosslinks. As the strain increased from 10% to 90%, the hydrogel network was disrupted and the material behaved like a liquid. When the strain returned to 10%, hydrogels rapidly reformed a gel state. Although there was some mechanical loss during the recovery, the gel-sol transitions were fully reversible over four consecutive alternating cycles, demonstrating the excellent self-healing behavior of PETyr and PETyrO hydrogels.

### *In vitro* photothermal effect of PETyrO hydrogel

**Figure 3:**
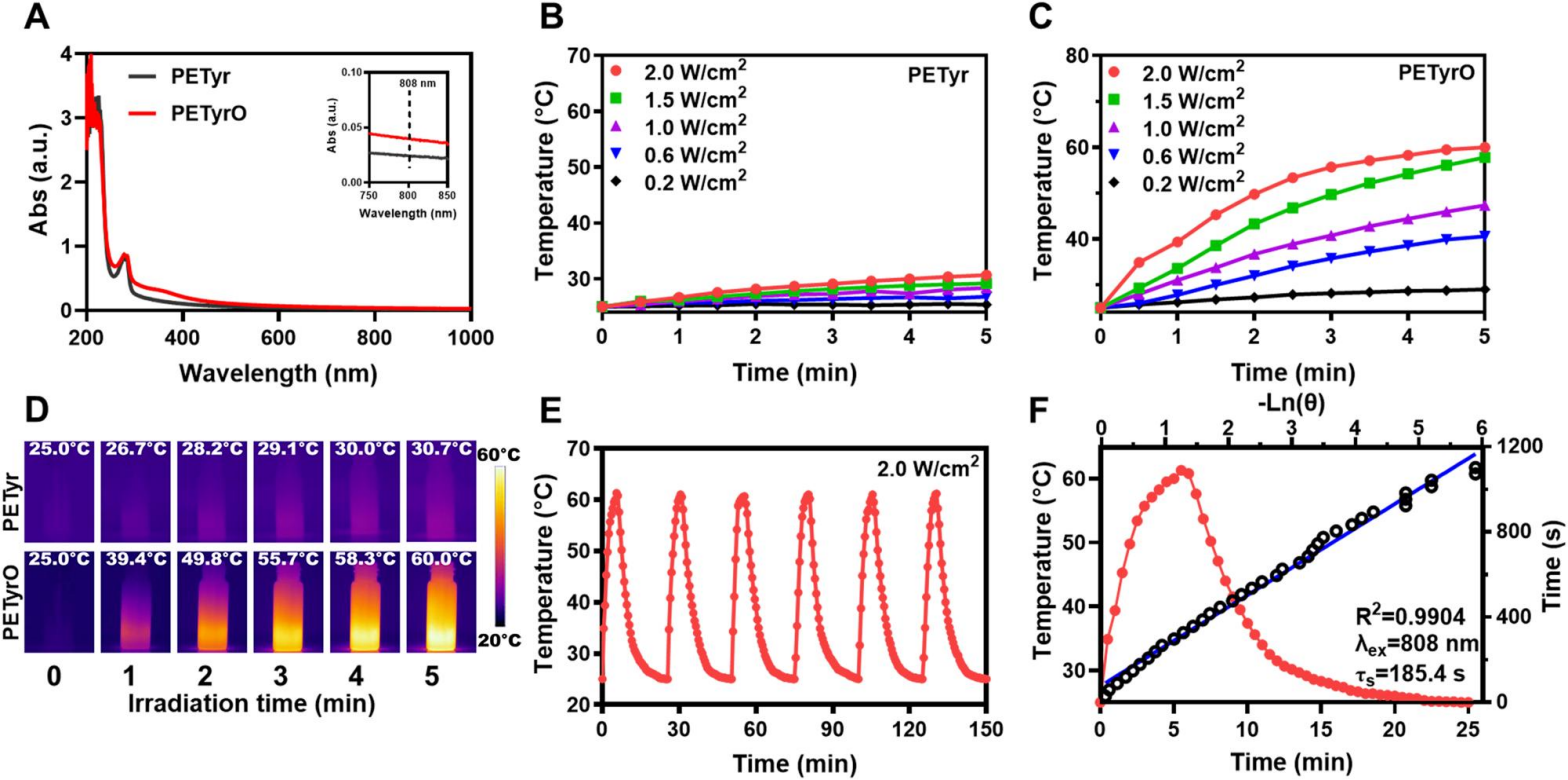
*In vitro* photothermal properties of PETyrO. (**A**) UV-vis absorption spectra of PETyrO. Temperature variation of PETyr (**B**) and PETyrO hydrogels (**C**) under 808 nm laser irradiation (0.2, 0.6, 1.0, 1.5, 2.0 W/cm^2^) for 5 min. (**D**) Time-dependent infrared thermal images of PETyr and PETyrO hydrogels under 808 nm irradiation (2.0 W/cm^2^) at 0–5 min intervals. (**E**) Cyclic photothermal stability of PETyrO under NIR laser irradiation (808 nm, 2.0 W/cm^2^). (**F**) Linear correlation between irradiation time and -ln(θ).

The melanin-inspired PETyrO hydrogel exhibits photothermal properties through efficient light absorption and conversion into heat. Notably, its optimized photothermal conversion efficiency highlights its potential for tumor therapy applications [40]. UV-vis spectrophotometric analysis revealed that PETyrO has significantly higher absorption at 808 nm than PETyr (Figure 3A). The photothermal conversion efficiency was then quantified. PETyrO hydrogels were prepared in deionized water at a concentration of 100 mg/mL and irradiated with an 808 nm near-infrared laser at varying power densities (0.2, 0.6, 1.0, 1.5 and 2.0 W/cm^2^) for 5 min. The PETyr hydrogel (Figure 3B) showed only a modest temperature increase (25°C to 30.7°C), even at 2.0 W/cm^2^, whereas the PETyrO hydrogel (Figure 3C) displayed a substantial photothermal response, reaching approximately 60°C after 5 min at 2.0 W/cm^2^. Temperature changes measured by IR thermography were consistent with those obtained by conventional thermometry (Figure 3D). To evaluate the photothermal stability, the PETyrO hydrogel underwent six on/off laser cycles (2.0 W/cm^2^, 5 min irradiation, followed by 25 min cooling). The hydrogel exhibited minimal temperature variation during prolonged irradiation, demonstrating robust photothermal stability (Figure 3E). The heating of PETyrO hydrogel scaled with power density, with the maximum 2.0 W/cm^2^ achieving ∼60°C within 5 min and maintaining consistent heating profiles over six cycles, suggesting potential for cancer cell ablation. Using established methodologies [41], the photothermal conversion efficiency of PETyrO was calculated to be 36%, which is in a favorable range for many PTT platforms.

The melanin-inspired PETyrO hydrogel demonstrates a pronounced photothermal response under 808 nm irradiation, with markedly higher absorbance at this wavelength than PETyr. In aqueous solution at 100 mg/mL, PETyrO heats rapidly under irradiation, achieving about 60°C within 5 min at the highest tested power density (2.0 W/cm^2^), whereas PETyr exhibits only modest heating under the same conditions. And six on/off laser cycles show that PETyrO maintains a robust, repeatable heating profile with minimal drift, indicating good photothermal stability. These findings suggest that PETyrO combines strong 808 nm absorption with efficient and stable photothermal conversion, supporting its potential for tumor ablation applications.

### *In vitro cytotoxicity* and antioxidant of PETyrO hydrogels

**Figure 4:**
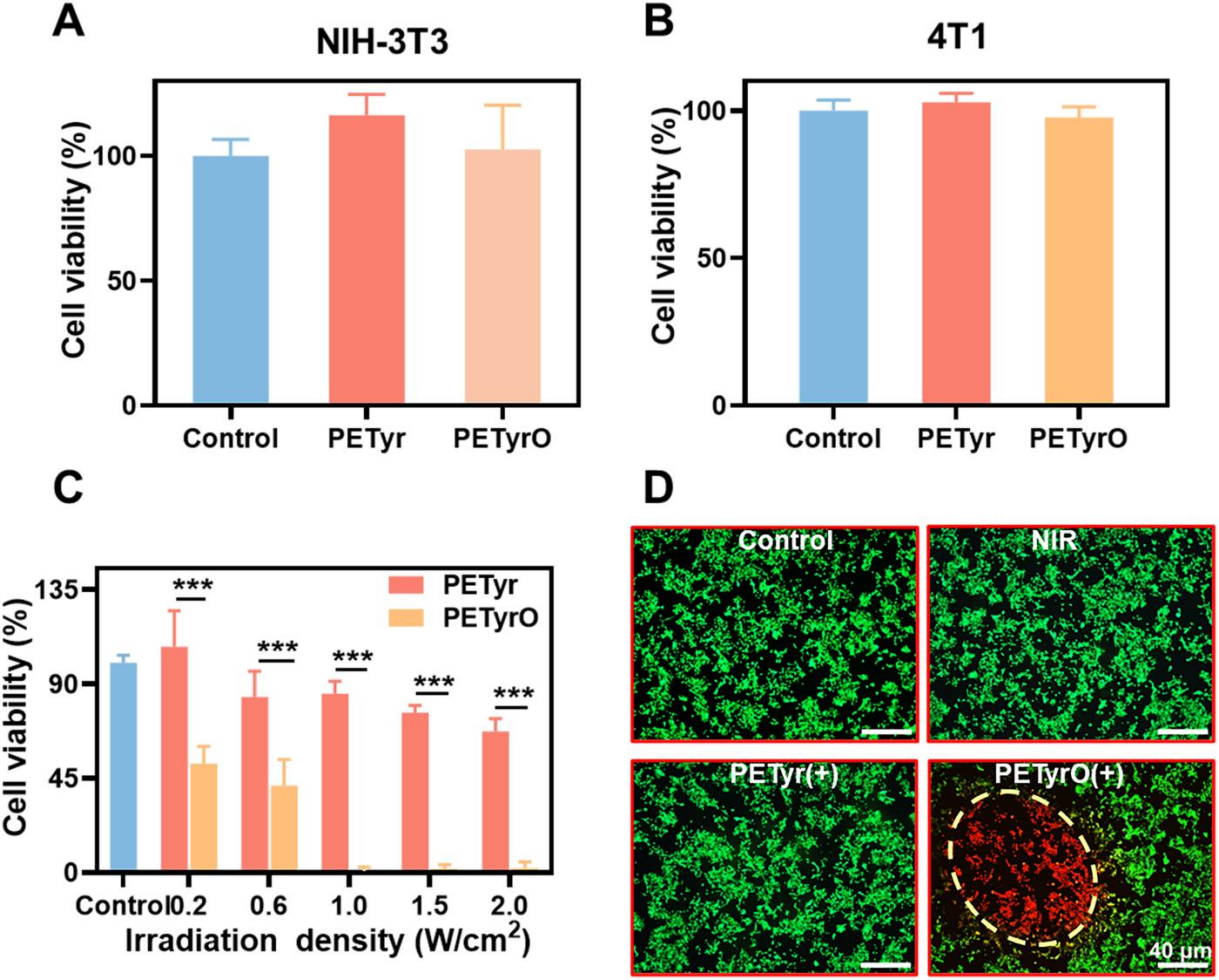
*In vitro* cytotoxic effects of PETyrO hydrogels. (**A**) NIH-3T3 and (**B**) 4T1 cell viability following treatment with PETyrO hydrogels (*n* = 3). (**C**) 4T1 cell viability after laser irradiation at increasing energy densities combined with PETyrO hydrogel treatment (*n* = 3). (**D**) Fluorescence micrographs showing 4T1 cells co-stained with calcein AM (green, live cells) and propidium iodide (red, dead cells) following laser irradiation. Data represent mean ± SD, ***P < 0.001, **P < 0.01, *P < 0.05. Scale bar: 40 μm.

The superior photothermal conversion efficiency of PETyrO hydrogel highlights its potential as a PTT agent for oncological treatment. The biocompatibility of PETyrO was first assessed. Figure 4A and B show that PETyrO exhibited no significant cytotoxicity toward either normal (NIH-3T3) or tumor (4T1) cell lines. Subsequent evaluation focused on its photothermal effects at the cellular level. 4T1 cells were used as *in vitro* tumor models. Cells were incubated with PETyr or PETyrO hydrogel for 30 min before irradiation with an 808 nm NIR laser at varying power densities (0.2, 0.6, 1.0, 1.5 and 2.0 W/cm^2^) for 5 min. Figure 4C reveals a laser-power-dependent decrease in cell viability within the PETyrO group. Notably, nearly complete cell death (approximately 100%) was achieved in the PETyrO group at irradiation densities above 1.0 W/cm^2^, confirming exceptional photothermal cytotoxicity. This efficacy was further corroborated by live/dead cell staining. Figure 4D illustrates distinct spatial segregation under laser exposure, with viable cells (green) and necrotic cells (red) in the PETyrO-treated region showing a sharp boundary (yellow dashed circle), wherein laser-exposed regions exhibited >90% cell death. In contrast, control groups (PBS, PETyr hydrogel or laser-only treatments) showed minimal cell death (<5%).

These data indicate that PETyrO hydrogel mediates strong photothermal cytotoxicity against 4T1 tumor cells under 808 nm irradiation, with viability decreasing in a laser power-dependent manner and near-complete cell death observed at irradiance ≥1.0 W/cm^2^. Live/dead staining corroborates this localized killing within the irradiated region, while control conditions (PBS, PETyr hydrogel, laser alone) show minimal cytotoxicity. Nevertheless, before translating these findings to *in vivo* applications, several critical considerations must be addressed, particularly regarding the blood compatibility and overall *in vivo* safety of the PETyrO hydrogel, which warrant thorough evaluation.

### *In vivo* photothermal therapy effect of PETyrO hydrogel

Biocompatibility and biodegradability are critical for *in vivo* applications. Based on our previous research, polypeptide hydrogels can gradually degrade *in vivo* over time, reducing the risk of prolonged retention in the body [31, 32]. Subsequently, we evaluated the hydrogel biocompatibility through hemolysis assays and a subcutaneous implantation study prior to cancer therapy. As shown in Supplementary Figure S2A, the 5 mg/mL hydrogels did not cause hemolysis of red blood cells. Neither PETyr nor PETyrO induced erythrocyte damage at 5 mg/mL, which is higher than the 1 mg/mL concentration used in cell viability assays. Histopathological analyses after 4 weeks of subcutaneous implantation revealed no tissue damage-no myocardial necrosis, alveolar septal thickening or other structural abnormalities across experimental groups, supporting the material’s biocompatibility (Supplementary Figure S2B).

**Figure 5:**
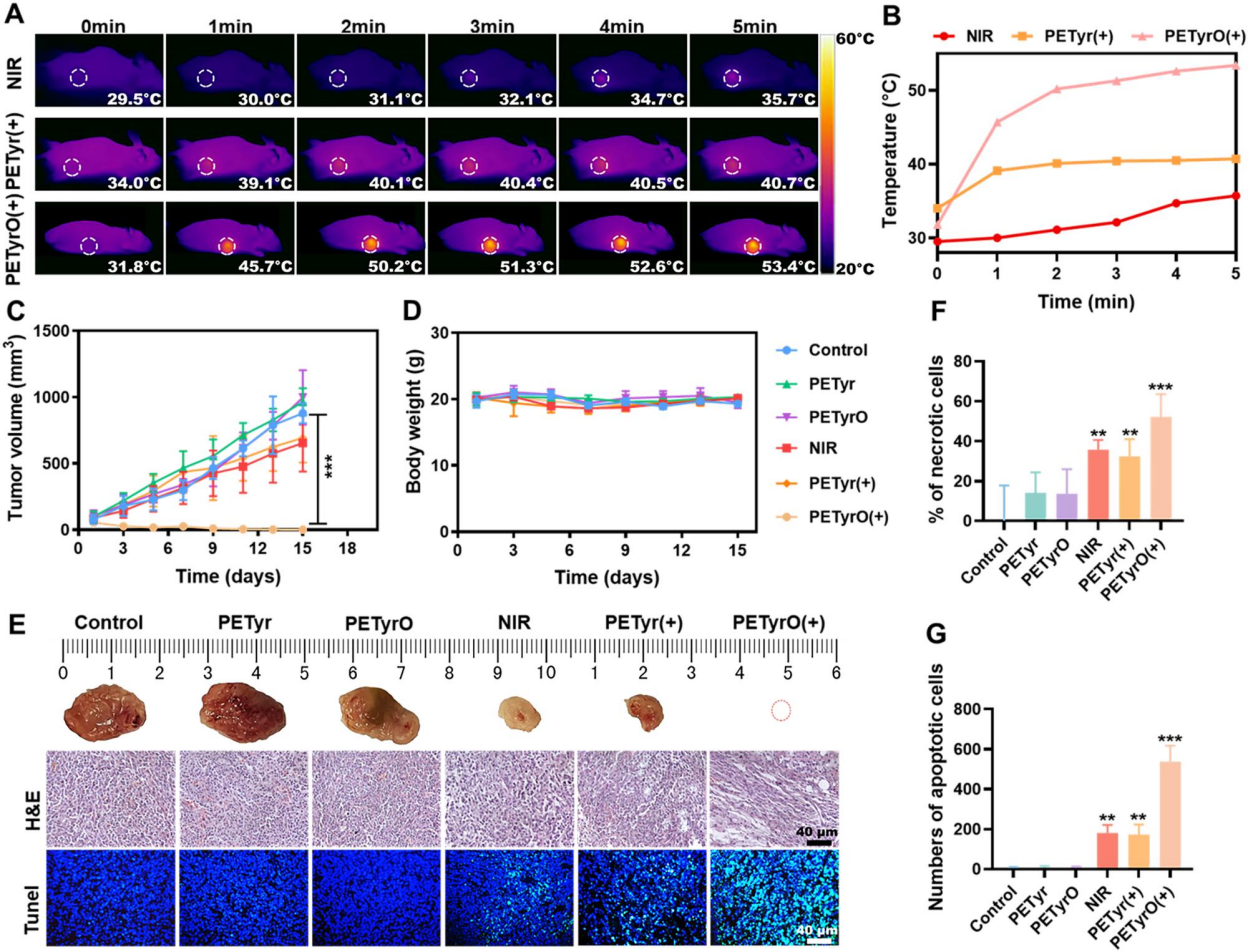
*In vivo* inhibition of tumor growth using PETyrO hydrogel. (**A**) The time-dependent *in vivo* infrared thermal imaging of tumors. (**B**) Temperature changes over time following laser irradiation. (**C**) Relative tumor volume and (**D**) Body weight measurements in 4T1 tumor-bearing mice following different treatments. (**E**) The representative digital image of tumors, H&E staining and TUNEL assay. (**F**) Quantification of necrotic cell percentage. (**G**) Quantification of apoptotic cell counts. Data represent mean ± SD (*n* = 6), ***P < 0.001, **P < 0.01, *P < 0.05. Scale bar: 40 μm.

Following the demonstrated ability of hydrogels to enhance cytotoxicity through PTT-induced heat stress *in vitro*, we further investigated their tumor growth inhibition efficacy *in vivo* using a 4T1 breast cancer mouse model. In tumor-bearing mice, hydrogels were subcutaneously administered near the tumor to assess local antitumor efficacy. The experiment included six groups: PBS, PETyr and PETyrO (no irradiation); PETyr (+) and PETyrO (+) (808 nm laser irradiation for 5 min) and an NIR control group (laser only). Infrared thermal imaging was recorded at 1-min intervals during irradiation (0–5 min). As shown in Figure 5A, PETyrO hydrogel achieved a rapid temperature rise to 53.4°C within 5 min, whereas the NIR group and the PETyr hydrogel reached only 35.7°C and 40.7°C, respectively, indicating superior photothermal conversion by PETyrO under physiological conditions. The PETyrO (+) group maintained temperatures above 50°C for extended periods (Figure 5B), supporting its potential for localized photothermal tumor therapy. Moreover, the PETyrO (+) treatment significantly suppressed tumor growth compared with controls, while all control groups exhibited progressive growth with no detectable inhibition (Figure 5C). Body weights remained stable across groups, suggesting minimal systemic toxicity (Figure 5D). By day 15, tumor volumes in the PETyrO (+) group approached negligible levels, with a statistically significant difference versus controls. Histological analysis of resected tumors (Figure 5E) showed complete tumor regression in the PETyrO (+) group, whereas substantial tumor mass persisted in other groups. Quantitative assessments of H&E and TUNEL staining revealed markedly increased necrosis and apoptosis in the PETyrO (+) tumors (Figure 5F and G). Collectively, these *in vivo* findings demonstrate potent photothermal antitumor efficacy of the PETyrO hydrogel with acceptable local tolerance, supporting its potential for localized PTT. Future work will address detailed degradation kinetics, comprehensive systemic safety assessments and mechanistic investigations to further validate translational potential.

### Antioxidant activities of PETyrO hydrogel *in vitro*

**Figure 6:**
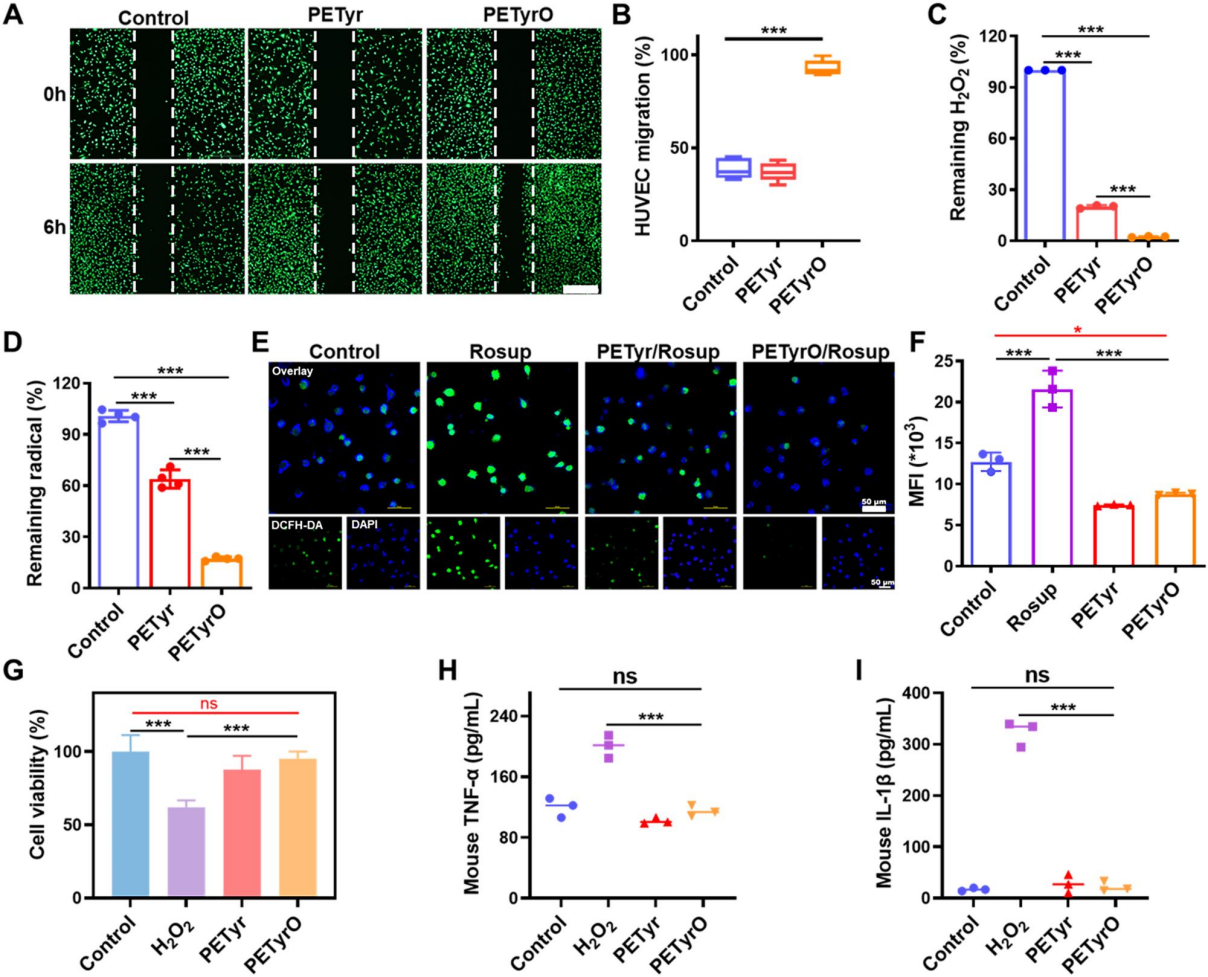
*In vitro* antioxidant activity of PETyrO hydrogel. (**A**) Representative images and (**B**) quantitative analysis of cell scratch assay (*n* = 3), scale bar: 500 μm. (**C**) *In vitro* H_2_O_2_ scavenging capacity of PETyrO hydrogel (*n* = 3). (**D**) Free radical scavenging activity of PETyrO hydrogel (*n* = 3). (**E**) Representative fluorescence micrographs of rosup-stimulated HUVECs, scale bar: 50 μm. (**F**) Intracellular ROS of rosup-stimulated HUVECs following PETyrO hydrogel treatment (*n* = 3). (**G**) Cell viability after H_2_O_2_ exposure (25 μg/mL) (*n* = 3). (**H**) TNF-α and (**I**) IL-1β secretion levels in macrophage culture supernatants (*n* = 3). ***P < 0.001, **P < 0.01, *P < 0.05.

Endothelial cell migration is a critical regulatory step in angiogenesis and vascular repair [42]. Accordingly, we systematically evaluated the pro-migratory capacity of the PETyrO hydrogel. PETyrO markedly enhanced HUVEC migration relative to the control group (Figure 6A and B). This effect is likely due to quinone-mediated adsorption of fibronectin onto the hydrogel surface, which promotes cell adhesion and cytoskeletal rearrangements through mechanotransduction pathways [43, 44]. To substantiate this mechanism, further experiments are needed, including direct assessment of surface fibronectin adsorption, interrogation of integrin- and FAK/Src-mediated signaling and differentiation between migration and proliferation contributions.

Furthermore, numerous phenolic hydroxyl-containing compounds, including polyphenolic polymers, polysaccharides, peptides and polyamino acids, exhibit potent ROS scavenging capacities [45]. To assess the antioxidant potential of PETyrO hydrogels, we measured their ability to scavenge H_2_O_2_ and free radicals. As depicted in Figure 6C and D, both PETyr and PETyrO hydrogels demonstrated scavenging activity toward H_2_O_2_ and free radicals, confirming the antioxidant properties of PETyr-based hydrogels. At a concentration of 100 mg/mL in a 50 μL volume, PETyr hydrogel exhibited an H_2_O_2_ scavenging rate of ∼80% and reduced nitrogen radical levels to ∼60%, significantly outperforming the control group. Notably, PETyrO hydrogel showed enhanced scavenging efficiencies of 97% for H_2_O_2_ and 82% for nitrogen radicals, further demonstrating the ROS-modulating capacity of these hydrogels. The hydrogels’ ability to scavenge intracellular ROS was assessed using the fluorescent probe DCFH-DA. Rosup-treated cells served as positive controls to induce elevated intracellular ROS levels. Figure 6E reveals that Rosup-stimulated HUVECs displayed markedly stronger DCF fluorescence signals than untreated controls. Compared with controls, PETyr-based hydrogel treatment significantly attenuated fluorescence signals, indicating effective intracellular ROS regulation. Flow cytometry analysis corroborated these findings, showing significant reduction in cellular fluorescence intensity following hydrogel incubation, consistent with intracellular ROS suppression (Figure 6F).

A CCK-8 assay was performed to evaluate the protective and anti-inflammatory effects of PETyrO hydrogel on H_2_O_2_-induced cellular damage. Exposure to 100 mM H_2_O_2_ reduced cell viability to approximately 60% (Figure 6G). In contrast, co-incubation with PETyrO hydrogel restored viability to over 90%, indicating its cytoprotective effect against oxidative stress. Analysis of pro-inflammatory cytokines showed that TNF-α and IL-1β levels in the PETyrO group were significantly lower than those in the H_2_O_2_-treated group and did not differ statistically from the control group (Figure 6H and I). These findings confirm that PETyrO hydrogel exhibits ROS-scavenging capacity and suggest its potential to mitigate thermal injury by neutralizing excess reactive oxygen species and promoting wound repair. PETyrO hydrogels display a dual ability to promote endothelial migration and modulate ROS, which could be advantageous for vascular repair and anti-oxidative protection, further facilitating wound healing.

### *In vitro* macrophage polarization

**Figure 7:**
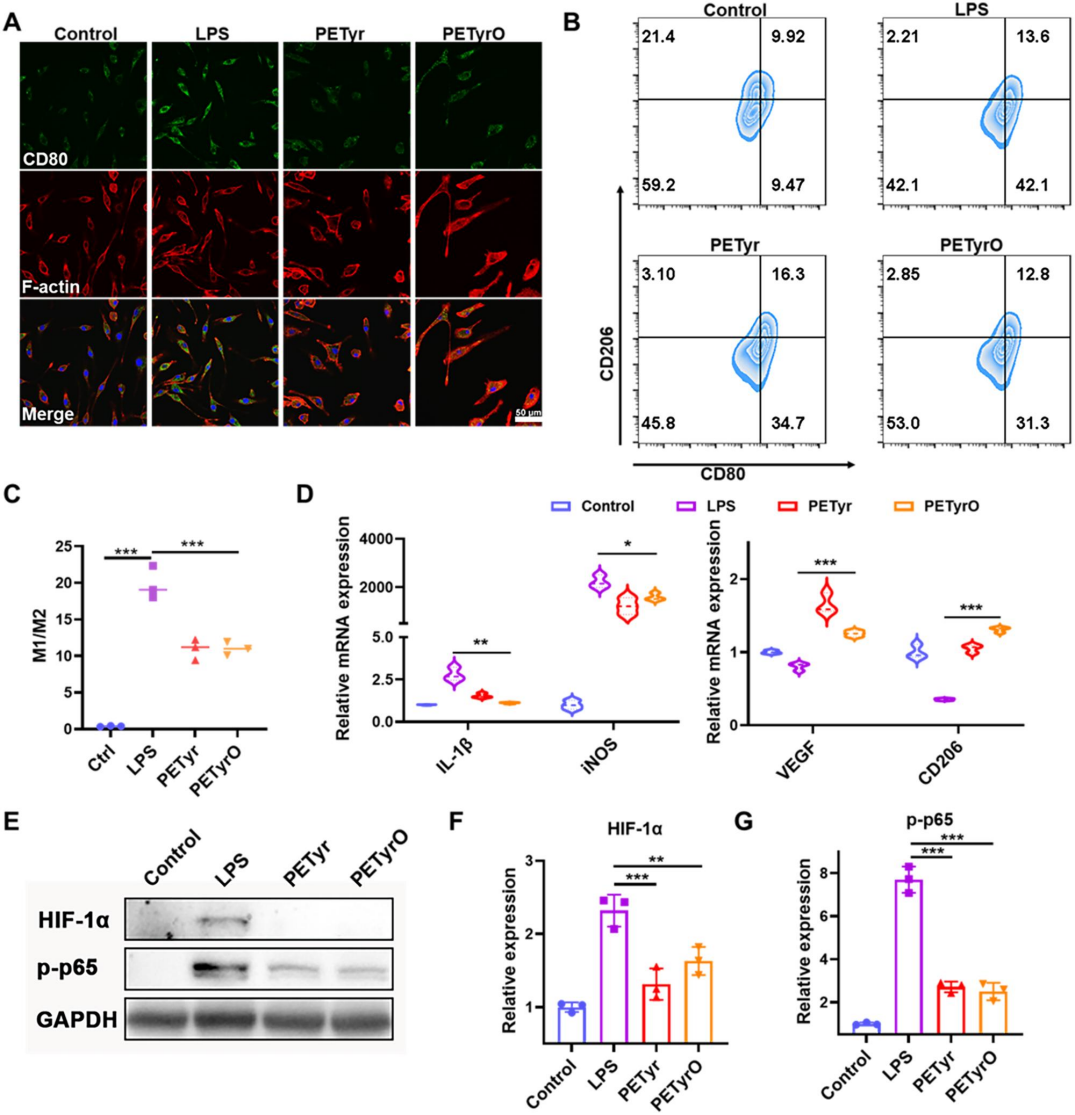
Regulation of macrophage polarization by PETyrO. (**A**) CLSM images of BMDMs treated with LPS or LPS/PETyrO for 48 h. CD80 (green), F-actin (red), nuclei (blue), scale bar: 50 μm. (**B**) Flow cytometric analysis of CD80 and CD206 expression in F4/80^+^ macrophages. (**C**) Quantification of M1/M2 macrophages, (*n* = 3). (**D**) Relative mRNA levels of IL-1β, iNOS, VEGF and CD206 (*n* = 3). (**E**) Western blot analysis of HIF-1α and phosphorylated p65 (p-p65) protein expression in macrophages. Densitometric quantification of (**F**) HIF-1α and (**G**) p-p65 protein bands (*n* = 3). ***P < 0.001, **P < 0.01 or *P < 0.05.

ROS act as regulators of multiple phases in wound healing. Following tissue injury, immune cells release signaling factors that initiate inflammatory responses in oxidative environments. Notably, the balance between M1 and M2 macrophage polarization determines cellular and tissue outcomes, with prolonged M1 dominance exacerbating tissue damage. Given PETyrO’s demonstrated ROS-scavenging capacity and cytoprotective effects against oxidative stress, we evaluated its regulatory impact on macrophage polarization using BMDMs. BMDMs cultured in standard medium served as negative controls, while lipopolysaccharide (LPS)-stimulated BMDMs served as positive controls for M1 activation. As shown in Figure 7A, 24 h after LPS stimulation, BMDMs displayed increased CD80 expression, indicating successful M1 polarization and inflammatory activation. Subsequent 48-h co-incubation with PETyrO significantly reduced CD80 expression compared to LPS treatment alone. Flow cytometry further validated M1 polarization: PETyrO treatment reduced CD80 levels by 25.7% relative to LPS-treated controls (Figure 7B). Quantitative analysis of M1/M2 phenotype ratio by flow cytometry showed a significant decrease following PETyrO treatment (LPS vs PETyrO: 19.8 vs 11.0) (Figure 7C). We further assessed inflammation- and repair-related gene expression by qRT-PCR (Figure 7D). In contrast to LPS-treated controls, the PETyrO group exhibited downregulation of M1-associated genes (IL-1β, iNOS) and concomitant upregulation of M2 markers (VEGF, CD206).

The transcription factor hypoxia-inducible factor-1 (HIF-1) acts as a master regulator of adaptive responses to hypoxia. Concurrently, NF-κB, a key mediator of inflammatory responses, modulates HIF-1α upregulation. Recent studies indicate that ROS promote HIF-1α mRNA transcription through activation of the NF-κB pathway [46, 47]. We therefore assessed both NF-κB signaling and HIF-1α expression. As shown in Figure 7E and Supplementary Figure S3, PETyrO treatment significantly suppressed both HIF-1α expression and NF-κB p65 phosphorylation. Quantitative analysis (Figure 7F and G) demonstrated a 29.7% reduction in HIF-1α levels (1.63 ± 0.19 vs 2.32 ± 0.22) and a 67.5% decrease in NF-κB p65 phosphorylation (2.50 ± 0.40 vs 7.70 ± 0.61) following PETyrO treatment relative to LPS controls. These findings suggest that PETyrO attenuates inflammation by suppressing HIF-1α expression *via* NF-κB signaling, though the precise mechanisms warrant further investigation.

Macrophage-regulated ROS play pivotal roles in wound healing, where the balance between pro-inflammatory M1 and pro-repair M2 macrophages shapes tissue outcomes. Our data show that PETyrO hydrogel markedly attenuates LPS-induced M1 polarization of BMDMs and promotes a shift toward an M2-like phenotype, as evidenced by reduced CD80 expression and downregulation of M1 markers (IL-1β, iNOS) with concurrent upregulation of M2 markers (VEGF, CD206). These effects were accompanied by suppression of NF-κB p65 phosphorylation and decreased HIF-1α levels, consistent with ROS scavenging dampening NF-κB signaling and downstream hypoxic responses. Collectively, PETyrO appears to mitigate inflammatory activation while enhancing repair-associated pathways.

### *In vivo* wound healing

Building on the *in vitro* experimental findings, we investigated the therapeutic potential of PETyrO hydrogels in accelerating wound healing through ROS regulation using a thermal injury model. Supplementary Figure S4A demonstrates complete tumor eradication by week 3, with no observed recurrence or metastasis through week 4. Following photothermal therapy, characteristic thermal burns were observed on the murine epidermal surface. H&E staining (Supplementary Figure S4B) revealed severe structural compromise in both epidermal and dermal layers post-photothermal intervention. The complex *in situ* photothermal wound progression was monitored through serial measurements with photographic documentation at Days 0, 3 and 8. Histological evaluation using H&E staining was performed to characterize tissue regeneration. The mice in the PETyrO (+) group that underwent photothermal treatment were randomly assigned to two subgroups: PETyrO dressing treatment on the skin surface or no additional treatment (Control). Supplementary Figure S5 demonstrated accelerated healing in PETyrO-treated mice, with near-complete wound closure by day 8, in contrast to control mice that retained unhealed areas. H&E staining revealed that by day 3, scabs had fallen off in the PETyrO-treated group, with signs of dermal regeneration. By day 8, epidermal regeneration was evident in the PETyrO-treated group, while complete healing had not yet occurred in controls.

**Figure 8:**
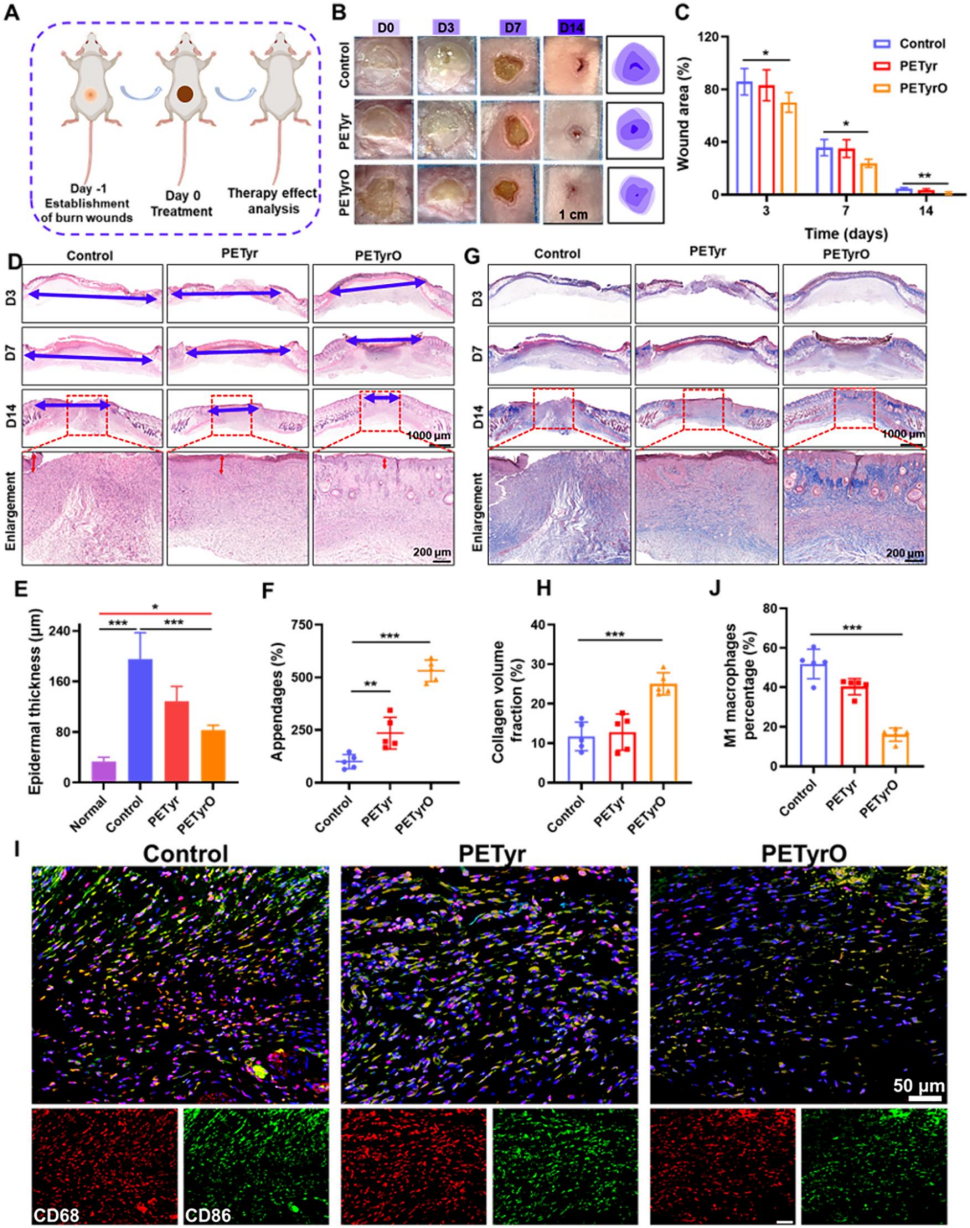
Burn wound healing accelerated by PETyrO hydrogel in mice. (**A**) Schematic diagram of burn wound treatment and experimental timeline. (**B**) Representative wound photographs and closure contours at postoperative days 0, 3, 7 and 14. (**C**) Quantification of wound area reduction rates. (**D**) H&E staining of wound sections on days 3, 7 and 14 post-injury. Purple arrows demarcate wound margins; red arrows indicate epidermal regeneration. (**E**) Epidermal thickness quantification. (**F**) Skin appendage regeneration assessment. Both analyses conducted on day 14 across treatment groups. (**G**) Collagen deposition analysis by Masson’s trichrome staining at specified time points. (**H**) Collagen volume fraction quantification at day 14. (**I**) Immunofluorescence staining of CD68^+^ (macrophages, red) and CD86^+^ (M1, green) macrophages at day 7. (**J**) M1 macrophage polarization ratio quantification. Data presented as mean ± SD (*n* = 5). ***P < 0.001, **P < 0.01 or *P < 0.05.

Since the injected hydrogel was located near the tumor tissue beneath the skin, while our wound dressing was used for the epidermal layer. As time goes on and with the application of NIR irradiation in tumor treatment, there may be differences in the retention amount of the hydrogel. In order to eliminate the influence of the previously injected hydrogel, standardized heat-induced burn models with 0.8 cm diameter circular wounds were established, applying PETyrO hydrogel as a bioactive dressing. The experimental results are presented in Figure 8. Specifically, PETyrO hydrogel demonstrated superior wound repair and skin regeneration compared with the control group (Figure 8A). Quantitative analysis showed consistently smaller wound areas in the hydrogel-treated group across all observation time points (Figure 8B and Supplementary Figure S6). By day 14, PETyrO-treated wounds achieved near-complete healing, with a closure rate of 99.0% ± 1.1% (Figure 8C), indicating accelerated tissue regeneration. Collectively, these findings demonstrate that PETyrO hydrogel acts as an effective wound dressing capable of significantly accelerating the healing process.

Histopathological features of skin wound healing were analyzed using H&E and Masson’s trichrome staining. On post-wounding day 3, epidermal and dermal defects were observed across all groups (Figure 8D), indicating pronounced burn-related structural damage. Notably, the PETyrO hydrogel group exhibited a reduced wound edge length (purple arrows) compared with controls. By day 7, complete scab detachment occurred in all groups, with the PETyrO hydrogel group showing the initiation of dermal regeneration. By day 14, the PETyrO hydrogel group demonstrated near-complete tissue restoration, with epidermal thickness approaching normal and the shortest wound margins (Figure 8E). The distinct presence of numerous mature hair follicle structures in the PETyrO hydrogel-treated group confirmed epidermal layer regeneration (Figure 8F). Collagen deposition, assessed by Masson’s trichrome staining, showed that by day 14 the PETyrO hydrogel-treated group exhibited highly organized collagen fiber alignment and significantly increased fiber density compared with controls (Figure 8G and H), indicating enhanced extracellular matrix remodeling. Collectively, these findings demonstrate that PETyrO hydrogel accelerates burn wound healing through three synergistic mechanisms: (i) facilitating epidermal regeneration, (ii) enhancing dermal tissue formation and (iii) promoting collagen deposition.

To investigate the physiological mechanisms of wound repair, we employed immunofluorescence staining to assess the anti-inflammatory properties of PETyrO hydrogel dressings *in vivo*, focusing on macrophage infiltration and polarization within the wound subcutaneous tissue. CD68 immunostaining revealed substantial macrophage accumulation (red fluorescence) in the control group (Figure 8I), indicating persistent inflammatory activity after injury. Quantitative analysis showed a 69.1% reduction in CD68^+^CD86^+^ M1 macrophages in PETyrO hydrogel-treated wound tissues compared with controls (Figure 8J). Collectively, these findings indicate that the PETyrO hydrogel modulates local inflammatory responses, thereby accelerating wound healing progression. When combined with prior data on tumor PTT, PETyrO exhibits a dual functionality as a bioactive wound-dressing material that supports rapid tissue restoration while enabling localized photothermal therapy where applicable.

## Conclusions

In summary, we developed a synthetic, nontoxic and biodegradable PETyrO hydrogel *via* ROP of L-tyrosine NCAs. This system serves as a versatile platform for tumor therapy and wound healing. Leveraging structural and functional similarities to melanin, the oxidized PETyrO hydrogel demonstrates exceptional photothermal conversion efficiency (η = 36%) and stability, achieving nearly complete tumor ablation in murine models. Moreover, the hydrogel’s phenolic components effectively scavenged ROS, reducing thermal damage to peritumoral tissues and accelerating wound closure compared with controls. This biocompatible platform enables sequential cancer therapy and tissue regeneration: initial photothermal ablation (50°C tumor hyperthermia) followed by ROS-scavenging-mediated repair. In conclusion, the hydrogel will serve as a versatile, integrated platform for post-treatment management, enabling local tumor control *via* photothermal ablation while promoting rapid wound healing, antioxidation and anti-inflammatory responses in skin and soft tissue tumors and related settings.

## Funding

This work was supported by the National Natural Science Foundation of China (82272066), General Program of Jiangsu Health Commission (M2024103), Jinshan Talents in the Medical Field of Zhenjiang (JSYCBS202301) and the Basic Research Program of Zhenjiang City (JC2024027).

## Supplementary Material

rbag034_Supplementary_Data

## Data Availability

Data will be made available on request.
