# Supplementary material for: A dual-functional PEG-tyrosine hydrogel with photothermal effect and antioxidant capacity for cancer therapy and tissue regeneration
Source: Regen Biomater. 2026 Mar 4;13:rbag034. doi: 10.1093/rb/rbag034 (PMC13270973; doi:10.1093/rb/rbag034)
Supplement: rbag034_Supplementary_Data [file rbag034_supplementary_data.docx]

Supporting Information

**A Dual-Functional PEG-Tyrosine Hydrogels with Photothermal Effect and Antioxidant Capacity for Cancer Therapy and Tissue Regeneration**

Haitao Zhu ^a,b,1^, Yunfan Shen ^a,1^, Zhebin Wu ^a,1^, Yide Yi ^a^, Yuanfei Chen ^a^, Wenjin Xu ^a^, Xin Wang ^a^, Hsiang-I Tsai ^a,b^, Dongqing Wang ^a,b^, Xiang Liao ^a,*^, Yanfang Liu ^c,*^, Qinghua Li ^a,b,*^


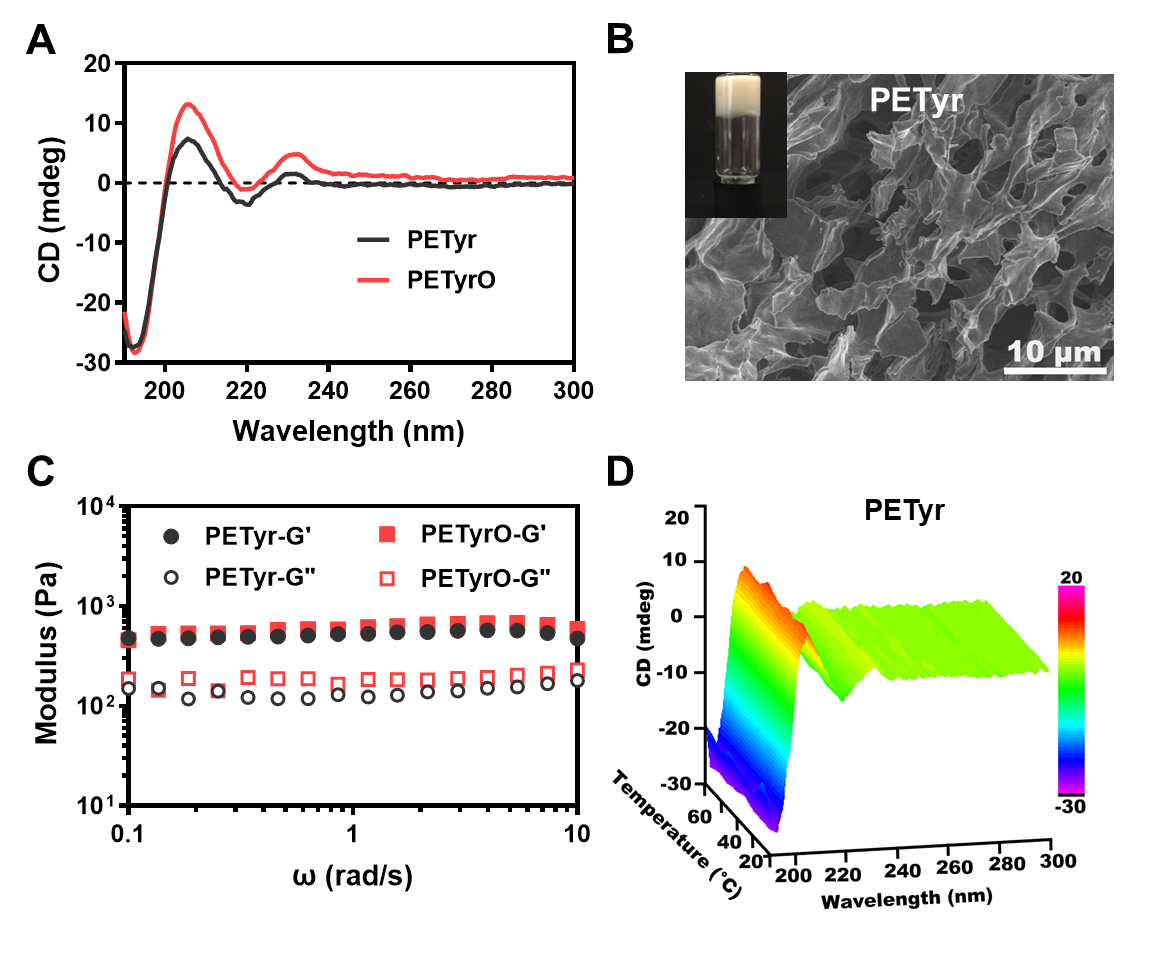


**Figure S1**. (A) CD spectra. (B) Interior microstructure of PETyr hydrogel observed by SEM. (C) Viscoelastic properties of the hydrogels: Elasticity modulus (G’) and viscosity modulus (G’’) of PETyrO hydrogel. (D) The CD spectra of PETyr hydrogel.


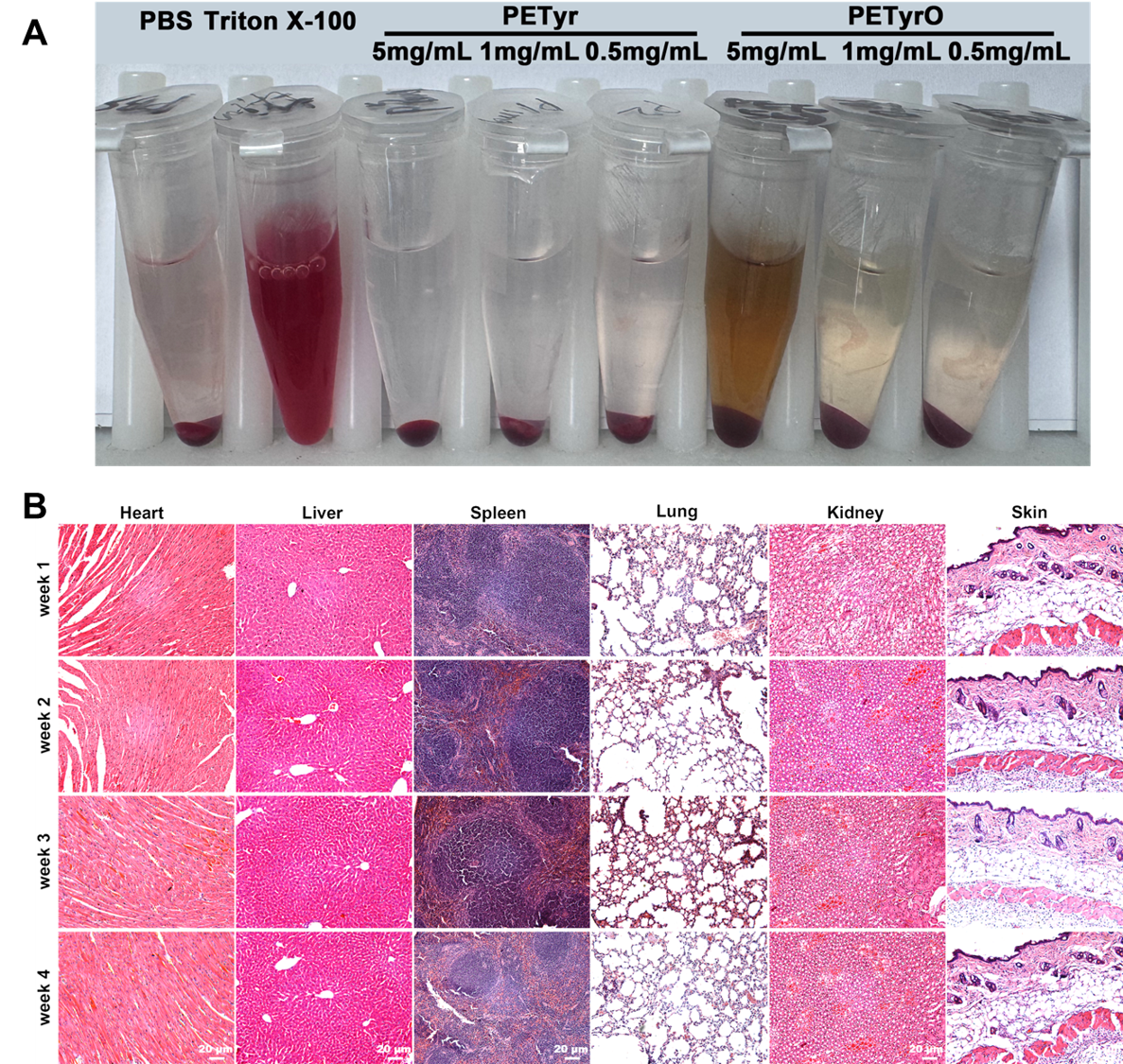


**Figure S2**. (A) Images of the hemolysis test. (B) H&E staining after four weeks of subcutaneous implantation of PETyrO hydrogel.


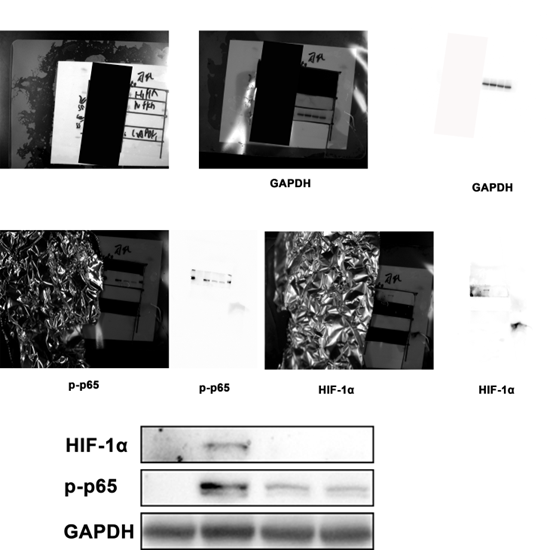


**Figure S3**. The original, uncropped Western blot images.


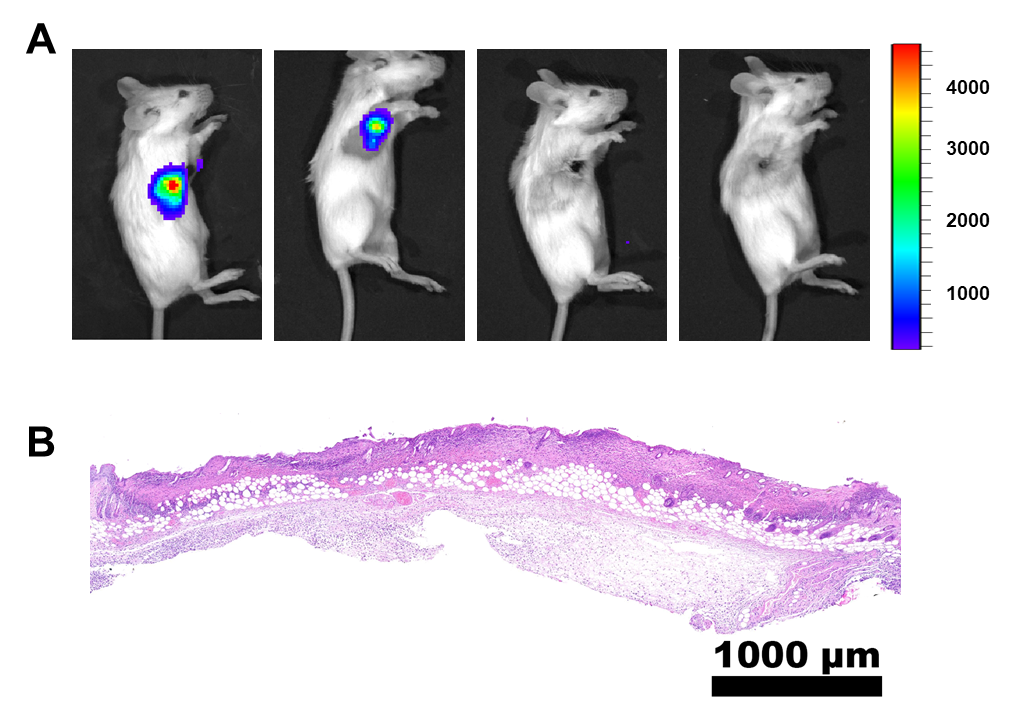


**Figure S4**. Damaged epidermis and dermis of the skin after the photothermal treatment.


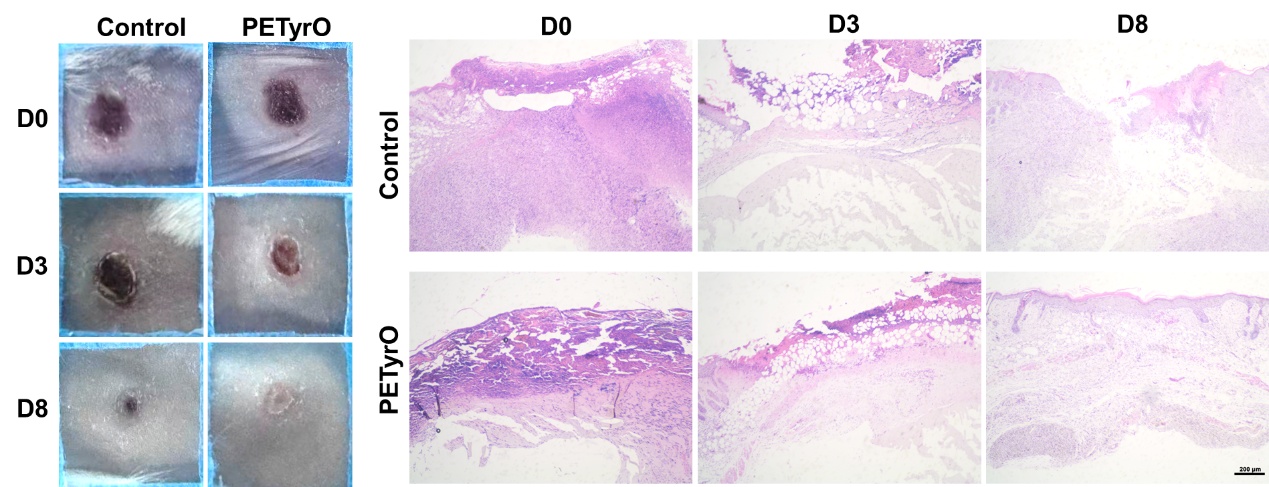


**Figure S5**. Thermal injury wounds healing accelerated by PETyrO hydrogel in mice after photothermal therapy for tumors (scale bar: 200 μm).


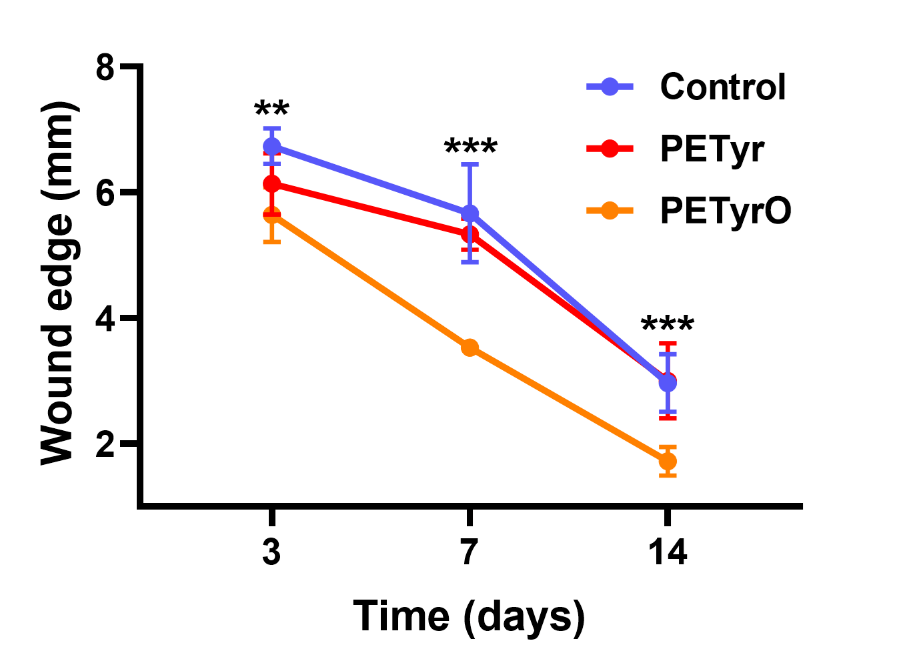


**Figure S6**. Wound edges with trearment at different time.
